# The Role of Nutritional Factors in the Modulation of the Composition of the Gut Microbiota in People with Autoimmune Diabetes

**DOI:** 10.3390/nu14122498

**Published:** 2022-06-16

**Authors:** Anna Winiarska-Mieczan, Ewa Tomaszewska, Janine Donaldson, Karolina Jachimowicz

**Affiliations:** 1 Department of Bromatology and Nutrition Physiology, Institute of Animal Nutrition and Bromatology, University of Life Sciences in Lublin, Akademicka St. 13, 20-950 Lublin, Poland; karolina.jachimowicz@up.lublin.pl; 2 Department of Animal Physiology, Faculty of Veterinary Medicine, University of Life Sciences in Lublin, Akademicka St. 12, 20-950 Lublin, Poland; 3 School of Physiology, Faculty of Health Sciences, University of the Witwatersrand, 7 York Road, Parktown, Johannesburg 2193, South Africa; janine.donaldson@wits.ac.za

**Keywords:** type 1 diabetes mellitus, tea, herbs, probiotics, prebiotics, postbiotics, synbiotics

## Abstract

Type 1 diabetes mellitus (T1DM) is a disease marked by oxidative stress, chronic inflammation, and the presence of autoantibodies. The gut microbiota has been shown to be involved in the alleviation of oxidative stress and inflammation as well as strengthening immunity, thus its’ possible involvement in the pathogenesis of T1DM has been highlighted. The goal of the present study is to analyze information on the relationship between the structure of the intestinal microbiome and the occurrence of T1DM. The modification of the intestinal microbiota can increase the proportion of SCFA-producing bacteria, which could in turn be effective in the prevention and/or treatment of T1DM. The increased daily intake of soluble and non-soluble fibers, as well as the inclusion of pro-biotics, prebiotics, herbs, spices, and teas that are sources of phytobiotics, in the diet, could be important in improving the composition and activity of the microbiota and thus in the prevention of metabolic disorders. Understanding how the microbiota interacts with immune cells to create immune tolerance could enable the development of new therapeutic strategies for T1DM and improve the quality of life of people with T1DM.

## 1. Introduction

Type 1 diabetes mellitus (T1DM) is an autoimmune disease characterized by insufficient insulin production due to the destruction of the insulin-secreting β cells in the islets of Langerhans of the pancreas [1]. In addition to antigen specificity, an important factor influencing the destruction of pancreatic β cells is the quality of the immune response to islet cell antigens [2]. The genetic factor is crucial in the development of T1DM (most often the presence of human leucocyte antigens HLA, DR3-DQ2, and DR4-DQ8), but environmental factors, including nutrition, stimulate the initiation and progression of the disease [3,4]. T1DM is characterized by disturbances in the efficiency of the antioxidant system, progressive inflammation, and the presence of autoantibodies [3]. In recent years, the number of T1DM cases has been increasing and some cases cannot always be explained by genetic factors [5]. It has been suggested that lifestyle changes, including the frequent use of antibiotics, may be responsible for the prevalence of T1DM, through the modification of the gut microbiome composition [5,6]. Therefore, in recent years, a relationship has been suggested between the structure and composition of the gut microbiota and the risk of T1DM development [7]. Studies in laboratory animals and clinical observations in humans with T1DM have shown changes in the number of bacteria, including *Firmicutes*, *Bacteroides*, *Clostridium* cluster, *Lactobacillus*, *Bifidobacterium*, and *Prevotella*, compared to that of healthy subjects [8], hence the suggestion that dysbiosis in various sections of the intestine may promote the development of T1DM by increasing intestinal permeability in predisposed individuals.

T1DM is a disease marked by oxidative stress, chronic inflammation, and the presence of autoantibodies [3]. The gut microbiota has been shown to be involved in the alleviation of oxidative stress [9] and inflammation [10], as well as strengthening immunity [11]; thus, its possible involvement in the pathogenesis of T1DM was highlighted. The intestinal commensal microbiota is a unique ecosystem associated with various functions of the living organism, especially those related to immunity. Dysbiosis of the intestinal microbiota plays a key role in the pathogenesis of autoimmune diseases [12]. Microbiome modulation is one of the most promising new strategies in medicine to improve the health of people with autoimmune diseases [12,13]. The goal of the present study is to analyze the information on the relationship between the structure of the intestinal microbiome and the occurrence of T1DM. The possibilities of supporting the intestinal microbiota by introducing certain food products into the diet, such as tea, herbs, and spices, as well as probiotics and prebiotics as methods of diet therapy supporting the pharmacological treatment of T1DM are also analyzed.

## 2. Materials and Methods

**Figure 1:**
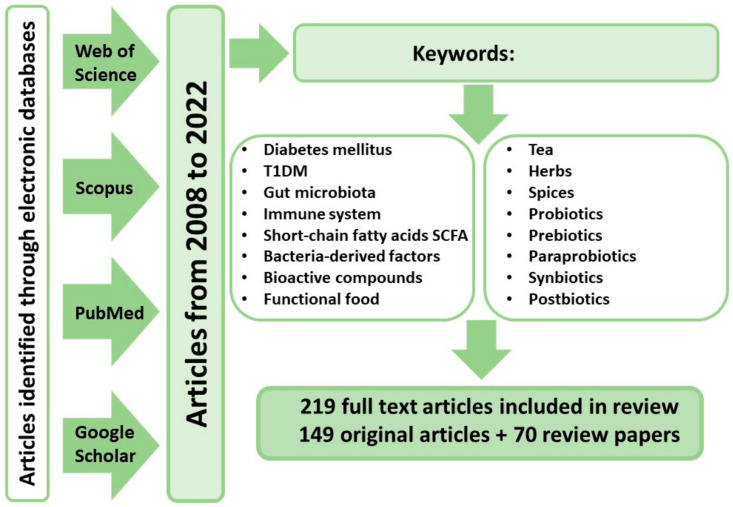
Research strategy employed in the review of available literature. T1DM—Type 1 diabetes; SCFA—Short-chain fatty acids.

The analysis of information available in the global scientific literature was conducted in August 2021 using the following databases: PubMed, Scopus, Google Scholar, and Web of Science. The databases were searched for both joint and separate instances of the keywords, e.g., “autoimmune diabetes”, “diabetes”, “T1DM”, “gut microbiota”, “tea”, and “herbs” in both Polish and English languages (Figure 1). Based on the titles and synopses, articles unrelated to the substantive criteria were excluded, and the remaining original and review papers were intensely analyzed to identify the most pertinent publications. Bibliographies were also reviewed in all the selected articles to identify other potentially viable texts. The search was narrowed to papers published between 2008 and 2022. Ultimately, a total of 2646 publications were reviewed, of which 219 were used: 149 research reports and 70 reviews.

## 3. The Importance of Microbiota for Human Health

Microbiota are essential for the proper functioning of the digestive tract since they regulate digestive processes by affecting the length and thickness of the intestinal villi [14]. The microbiota also influence the motor activity of the intestines playing a regulatory role in the intestinal metabolism of bile acids and serotonin [6]. Intestinal bacteria also have a positive effect on the growth, maturation, and proliferation of enterocytes, all of which influence the renewal of the epithelium. The intestinal bacteria adhere to receptors on the surface of the epithelium, sealing the intestinal wall and maintaining the integrity of the intestinal barrier [15]. By competing for nutrients, acidifying the intestinal environment, and synthesizing bacteriocins, the gut microbiota inhibit the development of pathogenic microorganisms and neutralize toxins and xenobiotics. They are also involved in the synthesis of some B vitamins and vitamin K [12,14,16,17,18]. Bolan et al. [19] confirmed a reduction in intestinal permeability to toxic metals (As, Cd, Pb, Hg) under the influence of intestinal microorganisms and chelating agents using an in vitro intestinal epithelial model from Caco-2 cells. A previous study on mice showed that some bacteria and their metabolites modulate the transcription of the intestinal epithelium in different ways [20]. Plasmocytes located in the intestinal wall, under the influence of certain microflora, secrete immunoglobulin A (IgA), which is key in the humoral immune response [21]. The IgA responses of the mucosa are devoid of the classic features of memory, which in turn enables the immune system of the mucosa to react to the constantly changing microbiota composition [22]. IgA reactions eliminate pathogens and promote host–microbial symbiosis [23].

The gut microflora performs many important functions and plays a key role in maintaining the proper homeostasis of the organism. Intestinal flora is very diverse, and the exact number of species is not fully known; it is estimated that there may be as many as 1500 different species of bacteria in the human digestive tract [24]. The gut microbiome encodes over 3 million genes, which is 100 times more than the number of human genes, so it is often referred to as the “second human genome” [12]. Characterizing the processes that govern the formation of the human microbiome is essential in understanding human development and physiology. Previous studies have shown that the profile of the placental microbiome is similar to that of the human oral cavity, which suggests that microbial colonization of the gastrointestinal tract begins in utero [11]. Both environments are dominated by *Firmicutes*, *Proteobacteria*, *Tenericutes*, *Bacteroidetes*, and *Fusobacteria* [25]. It has also been shown that meconium from full-term newborns is not sterile and that the bacteria present in it are also present in the amniotic fluid, vagina, and oral cavity [11]. Colonization also occurs at the time of childbirth: in the case of natural birth, the gastrointestinal tract of the newborn contains bacteria that live in the gastrointestinal tract and vagina of the mother, while in newborns born by caesarean section, the bacterial flora contains bacteria inhabiting the mother’s skin, so they have a smaller number of bacteria of the type *Bifidobacterium* and *Bacteroides* and more pathogenic *Enterococcus*, *Enterobacter*, and *Klebsiella* [26,27]. Following delivery, the diet is one of the main factors that modulates the gut microbiota of infants. In the first week of life, the intestinal microbiome of full-term infants is dominated by *Actinobacteria*, *Proteobacteria*, *Bacteroides*, and *Firmicutes* (to a lesser extent), while in premature babies these are mainly bacteria of the *Firmicutes* and *Tenericutes* genera, as well as *Actinobacteria* (in smaller numbers) [28]. The composition of the microbiome changes dynamically until it reaches a relatively mature equilibrium at about 3 years of age [25,29]. Until then, the structure of the microbiota is very unstable and reacts to changes in the diet, such as the introduction of solid foods or disease. The maturation of the gut microbiota early in human life induces enterocyte proliferation via microbial metabolites [15].

In adults, the groups of microorganisms show a clear differentiation in individual sections of the gastrointestinal tract, which is mainly related to the pH of the environment and the presence of oxygen. *Streptococcus*, *Peptococcus*, *Bifidobacterium*, *Staphylococcus*, *Lactobacillus*, and *Fusobacterium* predominate in the oral cavity, while *Helicobacter pylori*, *Lactobacillus*, *Streptococcus*, and *Candida albicans* are the most abundant in the stomach and duodenum. *Bacteroides*, *Lactobacillus*, and *Streptococcus* dominate in the jejunum, *Bacteroides*, *Clostridium*, *Enterococcus*, *Lactobacillus*, and *Veillonella* in the ileum, while *Firmicutes*, *Bacteroidetes*, *Proteobacteria*, and *Actinobacteria* predominate in the large intestine [14]. Changing the structure, abundance, and eubiotic state of the microbiota may contribute to intestinal and parenteral autoimmune and inflammatory disorders [12,30], which can lead to the occurrence of diseases associated with these conditions. It has been suggested that this condition could be the result of a lack of early stimulation of the immune system by biotic factors, which may interfere with the functioning of the immune system later in life and lead to hypersensitivity, autoimmune diseases, and/or inflammatory diseases [31]. This could explain the fact that there are more and more cases of autoimmune diseases, the occurrence of which cannot always be explained by genetic factors, but rather environmental factors, e.g., T1DM [5].

## 4. The Influence of Microbiota on the Immune System

The microbiome plays a key role in shaping and developing the main components of the host innate and adaptive immune system, while the immune system regulates the maintenance of the host–microbiota symbiosis [12,32]. Dysbiosis in a living organism, characterized by an overgrowth of facultative anaerobic bacteria and a decrease in diversity and balance, can induce an abnormal immune response that leads to a reduction in tolerance to the commensal microbiota. It can also interfere with the immune response, causing inflammation, oxidative stress, and insulin resistance [33].

An important function of the intestinal microbiota is the immuno-stimulation of cells of the lymphoid tissue associated with the intestine GALT (Gut-Associated Lymphoid Tissue), which is part of the immune system of the mucous membranes, MALT (Mucosa-Associated Lymphoid Tissue) [34]. About 70% of human lymphocytes, which are linked with intestinal GALT, which is made up of Peyer’s patches (sites of immune induction), mesenteric lymph nodes, and other lymphoid tissue (diffuse lymphoid regions not encapsulated) interact with autochthonous bacteria [34]. The modulation of the immune system by the microbiota begins even before birth, thanks to the microorganisms present in the amniotic fluid, which means that the fetus is exposed to bacterial antigens to which it must develop tolerance [29]. Following birth, diet and microbiota are factors that determine the proper maturation of the immune system, while the consumption of food, over and above supplying nutrients, also delivers antigens to the body [35,36]. Th infant’s immune system thus learns to control the balance between inflammatory and anti-inflammatory processes. The diversity of the gut microbiome during early colonization is critical to the formation of an immunoregulatory network that protects the body against mucosal IgE induction, which is associated with susceptibility to allergies [37]. The reactions initiated by commensal bacteria influence the antigen-specific adaptive immunity. Th17 lymphocytes (T-helper-17), a type of CD4 + T-helper cells, play a key role in coordinating the inflammatory immune response that provides protection against pathogens [38]. Th17 lymphocytes support actions against fungal and bacterial pathogens in the mucous membranes of the respiratory and digestive systems [38,39]. They limit side effects and ensure specific tolerance to food and bacterial antigens. The main pathological activity of Th17 lymphocytes is the maintenance of chronic inflammation, which contributes to the degradation of the body’s own tissues. The presence of Th17 has been demonstrated in allergic, autoinflammatory, and autoimmune diseases, including in people with T1DM [40,41,42]. Studies in mice have confirmed the role of intestinal bacterial metabolism in Th17 activation and in the development of Th17-dependent autoimmunity [43].

Undigested amino acids have the potential to become additional precursors for the production of SCFA by the gut microbiota; therefore, amino acids may have an indirect effect on the immune system [44]. Glycine, threonine, glutamine, and ornithine can be metabolized by anaerobic bacteria to produce acetate, while threonine, lysine, and glutamine can be used to synthesize butyrate [45]. One study has shown that the most abundant amino acid fermenting bacteria in the human small intestine are *Clostridia*, *Bacillus-Lactobacillus-Streptococcus*, *Proteobacteria*, and *Peptostreptococcus* [44].

Certain bacteria, such as *Faecalibacterium prausnitzii*, *Roseburia intestinalis*, and *Anaerostipes butyraticus*, produce short-chain fatty acids (SCFAs) during the fermentation of complex carbohydrates [46]. SCFAs, consisting mainly of butyrate, propionate, and acetate, by activating macrophages and dendritic cells, regulate the action of host immune cells by modulating the expression of proinflammatory cytokines, such as tumor necrosis factor-α (TNF-α), interleukin-12 (IL-12), and interleukin-6 (IL-6), as well as eicosanoids and chemokines [47]. SCFAs activate intestinal epithelial cells via G-protein coupled receptors (GPRs), GPR41 and GPR43, as shown in the studies in mice [47]. Kobayashi et al. [48] showed that GPR41 and GPR43 exert anti-inflammatory effects in human renal cortical epithelial cells. In addition, SCFAs inhibit the production of Th17 cells and promote the production of Treg lymphocytes (Regulatory T) from naive CD4 + T cells, which reduces inflammation [49,50]. Many autoimmune diseases result from an imbalance between pathogenic T cells and effector and regulatory T cells [51].

The intestinal microbiota stimulate the production of interleukin IL-10 by intestinal B cells through the activation of the TLR2/Myd88 (Toll-like receptor/myeloid differentiation factor 88 protein) signaling pathway, via PI3K (phosphoinositide 3-kinase) [52,53]. In addition to the initiation of B-cell differentiation, the microbiota stimulates the plasma cells that produce IL-10 and IgA+ [53]. In mice with a sterile digestive tract and with low-diversity microbiota, elevated serum IgE levels are found early in life [37]. This suggests that microbiota-derived immunoregulatory signals are essential in the maintenance of basal IgE levels, and that the proper induction of immune regulation requires adequate exposure to microbes early in life. Animals with a sterile digestive tract also have low levels of IgA in the gut and poorly developed lymphoid tissues associated with the gut, including small and sparse Peyer’s patches and mesenteric lymph nodes [54]. An efficient immune system is necessary to maintain a delicate balance between the elimination of pathogens and the maintenance of tolerance to one’s own tissues in order to avoid autoimmunity.

## 5. Pathogenesis of T1DM

T1DM is a chronic, genetically determined, autoimmune disease caused by the patient’s inability to secrete insulin as a result of autoimmune destruction of pancreatic β cells [3]. Pancreatic β-cell death is a result of the autoantibody-induced excessive production of proinflammatory cytokines, which in turn promotes phagocytosis, autophagy, and interferon activity. Pathological mechanisms, such as DNA methylation and modifications of histones and microRNAs, the action of which is related to the regulation of gene expression, are responsible for the development of T1DM [55]. About 95% of people with T1MD have the DR and DQ genes of the class-II human leukocyte HLA antigen (chromosome 6p21.3) or the UBASH3A mutation (chromosome 21q22.3), while 15% of people have an insulin-linked variable number of tandem repeats (INS-VNTR, chromosome 1p5,5), receptor cytotoxic T-Lymphocyte Antigen-4 (CTLA-4, chromosome 2q33), immune regulator (PTPN22, chromosome 1p13), and other genes [3,11].

In recent years, a relationship between the structure of the gut microbiota and the risk of T1DM has been suggested [5,8], which means that dysbiosis in various parts of the intestine may promote the development of T1DM by increasing intestinal permeability in predisposed individuals and lead to the occurrence of inflammation and oxidative stress. Moreover, T1DM is an autoimmune disease and the gut microbiota induces autoimmunity through TCR (T-cell receptors), thanks to which T cells recognize their antigens and are activated [51]. TCR have the ability to recognize many antigens (TCR cross-reactivity). One potential mechanism by which pathogenic autoreactive T cells may be activated is molecular mimicry, in which a foreign antigen exhibits significant structural or sequence similarity to its own antigen [51,56]. The activated autoreactive T cell can then enter the tissue containing its own antigen and trigger an autoimmune pathology. It is presumed that the development of autoimmunity is also influenced by metabolites and epigenetic modifications caused by the microbiota through the control of the immune system [57]. Bacterial metabolites can inhibit the production of inflammatory cytokines by β cells [58], and T cells that recognize β-cell antigens can be activated by bacterial products in the gut and then travel to the pancreatic lymph nodes to cause β-cell damage [59].

## 6. The Importance of Microbiota in T1DM

Increased intestinal permeability or ‘leaky gut’ is observed in many T1DM patients, which precedes clinical disease [51,60], as well as reduced microbiota diversity and an increased abundance of inflammatory species compared to healthy subjects [61]. A previous study on mice showed that the development of streptozotocin-induced T1DM (autoimmune diabetes) is dependent on the translocation of the intestinal microbiota into the pancreatic lymph node [62]. Moreover, the mice with T1DM were shown to have an altered intestinal microbiota composition, compared to that of control mice with an increase in the numbers of Bacteroides, Oscillospira (which disrupts the intestinal epithelial barrier), Sutterella, and Bifidobacterium [62]. Studies on the microbiome of children at risk of developing T1DM have shown a greater abundance of Bacterioides, Globicatella sanguinis, Dialister invisus, and Bifidobacterium longum and a reduction in the amount of Bifidobacterium pseudocatenulatum and Bifidobacterium adolescentis, compared to children without the burden of the disease [63,64]. In rats with T1DM, there was an increase in the number of pathogenic bacteria associated with infections and inflammation (*Ruminococcaceae*, *Shigella*, *Enterococcus*, *Streptococcus*, *Rothia*, *Alistipes*), while the number of beneficial SCFA-producing bacteria (*Lactobacillus*, *Faecalitalea*, *Butyricicoccus*) was reduced [65]. The microbiota of genetically predisposed infants, between the ages of 3 months and 3 years, are characterized by a greater number of Rikenellaceae, Ruminococcus, Streptococcus, and Blautia [61]. In addition, it was shown that the low abundance of lactate- and butyrate-producing strains was associated with the autoimmunity of β-cells in children, which may adversely affect the intestinal epithelial barrier function and stimulate inflammation [64].

The imbalance within the dominant genus, Bacteroides, is associated with the autoimmunity of the pancreatic islets, and the correlation analysis suggested a possible relationship between the signals of CrAssphage bacteriophages and the amount of Bacteroides dorei [66]. In this study, involving children who later developed islet autoimmunity, a lower number of Bacteroides vulgatus and Bifidobacterium bifidum was observed, compared to that observed in the control group. The study by Alkanani et al. [67], among people with islet autoimmunity, showed that changes in the gut microbiota correlate with susceptibility to T1DM. An increase in the relative abundance of Bacteroides and a decrease in the abundance of the anti-inflammatory, Prevotella, was observed in seropositive individuals with multiple antibodies, compared to those were only one autoantibody was found [67]. Similar results were obtained by Mejía-León et al. [29] when examining children with T1DM. In the microbiome of children with multiple islet auto-antibodies or confirmed T1DM, the anti-inflammatory species Prevotella and Butyricimonas were less frequent, and these differences were not related to diet. Moreover, these children had fewer SCFA-producing bacteria [60]. Giongo et al. [68] indicated that the high ratio of Firmicutes to Bacteroidetes and the instability of the microbiota may be one of the early diagnostic markers of the development of autoimmune disorders, including T1DM. Bedi et al. [69] found that changes in the gut microbiome in people with T1DM trigger the release of bacterial GAD (glutamic acid decarboxylase), thus injuring the host’s immune system due to the similarities between human and bacterial GAD. Talukdar et al. [70] showed that the composition of the gut microbiota differs significantly between people suffering from different types of diabetes: types 1, 2, and 3 (secondary pancreatogenic diabetes). The composition of the microbiome directly influences SCFA synthesis. Bacteroidetes mainly produce acetate and propionate, Firmicutes produce butyrate, and Bifidobacterium produce a large amount of acetate, as well as lactate, which is metabolized by other bacteria to butyrate [71]. This is why the large amount of Bacterioides contributes to an increase in intestinal permeability and thus to the development of autoantibodies against islet cells, which in turn leads to the translocation of microbes into the circulatory system, causing both direct damage and inflammation by immune damage of the pancreatic β cells [72].

## 7. Diet Components Positively Influencing the Development of Microbiota

Diet is a source of nutrients, but it is also the main route for the entry of antigens into the body. The intestinal microbiota metabolize proteins and complex carbohydrates, synthesize vitamins and produce a large number of metabolic products that can mediate contacts between the intestinal epithelium and cells of the immune system [33]. Questionable lifestyle and nutritional choices (albeit not always intentional), primarily in developed countries characterized by the West-type diet, may lead to the development of microbiota that lack the immunity and diversity necessary for a balanced immune response. It is believed that this may lead to an increase in autoimmune and inflammatory diseases [31,73]. Increased consumption of junk food has been shown to be associated with less gut microbiome diversity in children with T1DM [74]. The relationship between diet and immune response is largely regulated by the microbiota, as was shown in a study involving 482 healthy adult subjects [75].

### 7.1. Tea

Tea has positive effects on the health of people with T1DM due to the content of phenolic compounds that have antioxidant, anti-inflammatory, and immunomodulating properties [3]. At the same time, the presence of these compounds means that the consumption of tea can affect the composition of the intestinal microbiota by stimulating the growth of certain species or inhibiting the development of harmful species [76]. Green tea has been shown to have the strongest effect. Polyphenols are characterized by low bioavailability in the small intestine [77], therefore the participation of microbiota in their absorption is of great importance for human health. Most polyphenols are absorbed in the gut, but theaflavin and its galloyl derivatives, as well as teasinensin A, are more resistant to degradation by gut bacteria than most polyphenols [77,78]. Nevertheless, in vitro studies have shown that the microbiota plays an important role in the metabolism of theaflavins in both mice and humans. Lactobacillus plantarum 299v and Bacillus subtilis are of particular importance, as they metabolize the theaflavin-3,3′-digallate present in black tea [79].

Phenolic compounds show selective bactericidal activity. They damage bacterial cell membranes and thus inhibit the growth of Bacillus cereus, Campylobacter jejuni, Clostridium perfringens, Escherichia coli, Helicobacter pylori, Legionella pneumophila, and Mycobacterium spp. [80]. For example, epigallocatechin gallate (EGCG) can bind to peptidoglycans from the cell membranes of Gram-positive bacteria and cause them to break, while Gram-negative bacteria are protected from such effects by the outer membrane and negatively charged lipopolysaccharides that repel catechins [80].

Interactions between tea polyphenols and the gut microbiota lead to changes in the composition of the microbiota and the production of metabolites, including SCFAs, bile acids, and amino acids, which exert their biological effects both locally and systemically (Table 1). Glycans are formed as a result of the splitting out of glycosidic bonds in polyphenols. They are an important nutrient for the gut microbiota, especially for Bacteroidetes, as they have a greater ability to degrade glycans than Firmicutes [81]. By changing the composition of the microbiome and related metabolites, tea polyphenols may have a beneficial effect on the host organism, which contributes to the reduction in symptoms of some chronic metabolic disorders [82,83]. In vitro studies have shown that tea polyphenols promote the growth of Bacteroides, Faecalibacterium, Parabacteroides, and Bifidobacterium and inhibit Prevotella and Fusobacterium, and are therefore considered to be probiotic substances [76,77,84,85]. Flavonols can modulate the intestinal microbiota by affecting the adhesion of bacteria to enterocytes; this mainly applies to *Lactobacillus acidophilus* LA-5 and Lactobacillus plantarum IFPL379 [86]. Catechins inhibit the growth of certain pathogenic bacteria, such as Clostridium difficile and Staphylococcus spp., and may also stimulate the growth of beneficial Bifidobacterium bacteria [81]. Green tea extract, which is rich in catechins, especially EGCG, has been shown to improve the integrity of the intestinal barrier by reducing the translocation of gut-derived endotoxin and thus the resulting pro-inflammatory response in both animals and humans [87,88,89]. Campferol, which is present in tea leaves, improves the integrity of the intestinal barrier and inhibits inflammation in the intestines by reducing the activation of the TLR4/NF-κB pathway, and prevents the obesity-related gut dysbiosis [90].

Van Dorsten et al. [91] compared the microbial degradation of polyphenol-rich black tea extract and red wine extract in a five-step process, using an in vitro gastrointestinal model, which served as a simulator of the human intestinal microbial ecosystem. It was found that the production of phenolic acids and SCFAs by the gut microbiome was dependent on the region of the colon and on the source of the polyphenols. Tea extract inhibits the growth of Escherichia and Shigella, which are associated with gastrointestinal disorders, and increases the growth of Faecalibacterium, which synthesize butyrate [92]. A reduction in acetate synthesis was also found. A previous study by Unno and Osakabe [93] found that the administration of black or green tea extract to rats, at a dose of 10 g/kg, significantly modified the concentration of acetic and butyric acid observed in the cecum. It was found that black tea extract stimulates SCFA production more effectively than green tea extract, most likely by making it easier for the gut bacteria to use starch from the food consumed. In turn, the EGCG present in green tea was recognized as a potential modulator of the intestinal microbiota by stimulating an increase in the abundance of Akkermansia and the production of SCFAs in rats with colitis [94]. Green tea polyphenols have also been shown to lower the ratio of Bacteroidetes to Firmicutes and significantly increase the concentration of acetic and butyric acid in HFA mice [95]. Studies conducted on C57BL/6J mice showed a decrease in the number of Firmicutes and an increase in the number of Bacteroidetes in the caecum of the mice, due to polyphenols extracted from green and black tea, as well as an increase in SCFA synthesis correlated with an increase in the number of Pseudobutyrivibrio, which was especially pronounced in the groups receiving black tea [96]. Green, black, and oolong tea polyphenols inhibited the growth of Bacteroides, Prevotella and Clostridium histolyticum, and stimulated the growth of beneficial bacteria (Bifidobacterium, Lactobacillus, Enterococcus) in human Caco-2 cells [97]. The increased synthesis of SCFAs observed in this study was attributed to the microbial conversion of polyphenols in the pathways and processes occurring in the large intestine, which in turn stimulated the production of SCFA.

The administration of oolong tea polyphenols to obese mice for a period of four weeks caused an increase in the biodiversity of bacteria, with an increase in bacteria which produce butyrate and acetate, especially Bacteroidetes, and a simultaneous, favorable decrease in the proportion of Firmicutes and Bacteroidetes [84]. Guo et al. [85] highlighted the ability of oolong tea polyphenols to regulate the circadian rhythms of mice, by enhancing beneficial gut microbiota and influencing metabolic pathways by regulating gene expression. It is also possible that tea polyphenols influence the microbiome through the maintenance of an optimal redox status [98]. Lachnospiraceae, Bacteroides, Alistipes, and Faecalibaculum were identified as biomarkers of gut redox status in this study.

**Table 1:** Influence of tea on the microbiota composition.

	Design	Effects on Gut Microbiota	Effect on SCFA Level	References
Green tea	Post-menopausal females; four green tea pills/day for 12 months	=*Firmicutes*, =*Bacteroidetes*, =*Actinobacteria*	=acetic acid, = propionic acid, =butyric acid, =lithocholic acid, =deoxycholic acid	[99]
Green tea	C57BL/6J mice; 0.05, 0.2, or 0.8 g of green tea extract per 100 mL of water for 8 weeks	↓*Firmicutes*, ↑*Bacteroidetes*, ↑*Bacteroides*, ↑*Turicibacter*, ↑*Lachnospira*, ↓*Clostridium*	Not measured	[95]
Green tea	C57BL/6J obese mice, 0.5 g of decaffeinated green tea extract or black tea extract per 100 g of diet for 4 weeks	↑*Bacteroidetes*, ↓*Firmicutes*, ↓*Actinobacteria*, ↑*Parabacteroides*, ↑*Clostridium*, ↑*Coprococcus*, ↑*Pseudobutyrivibrio*	Green tea: = acetic acid, = propionic acid, = butyric acid, =valeric acid Black tea: = acetic acid, ↑propionic acid, ↑butyric acid, ↑valeric acid	[96]
Green tea	UVB-exposed Skh:HR-1 mice; diet containing 1% green tea extract for 10 weeks	↓*Lactobacillales*, ↑*Clostridia*, ↑*Erysipelotrichia*	Not measured	[100]
Green tea, black tea	Male Wistar rats; green tea extract 10 g/kg diet or black tea extract 10 g/kg diet for 3 weeks	Green tea: ↓*Prevotella*, ↓*Clostridium*, ↑*Lactobacillales*, =*Bifidobacterium*, =*Bacterioides*; Black tea: ↓*Clostridium*, =*Bifidobacterium*, =*Bacterioides*	Green tea: ↓acetic acid, ↓butyric acid; Black tea: ↑acetic acid, ↑propionic acid	[93]
Green tea, oolong tea, black tea	Caco-2 cells; 100 mL/well, green tea polyphenols, oolong tea polyphenols or black tea polyphenols at a final concentration of 25, 50, 100, and 200 lg/mL incubated for 24, 48, and 72 h, respectively	↑*Bifidobacterium*, ↑*Lactobacillus*/*Enterococcus*, ↓*Bacteroides*–*Prevotella*, ↓*Clostridium histolyticum*	↑acetic acid, ↑formic acid, ↑propionic acid, ↑butyric acid	[97]
Green tea, black tea, oolong tea	C57BL/6J obese mice, 100 mL tea infusion of green tea, oolong tea, or black tea in diet for 13 weeks	↑*Alistipes*, ↑*Rikenella*, ↑*Lachnospiraceae*, ↑*Akkermansia*, ↓*Bacteroides*, ↓*Parabacteroides*	Not measured	[101]
Pu-erh tea	Obese male Wistar rats; 750 mg/kg of ripe Pu-erh tea extract or 250 mg/kg of Pu-erh tea polyphenol or oxidized tea polyphenol for 12 weeks	↑*Firmicutes*, ↓*Bacteroidetes*, ↑*Actinobacteria*	Not measured	[102]
Pu-erh tea	Normal and overweight males; 50 mg/kg/day of instant Pu-erh tea infusion for 4 weeks	↓*Bacilli*, ↓*Clostridia*, ↓*Lactobacillus*, ↓*Bacillus*, ↓*Streptococcus*, ↓*Lactococcus*	Not measured	[103]
	C57BL/6J male mice; Pu-erh tea infusion (450 mg/kg/day) or 1.5 mg/mL theabrownin infusion (225 mg/kg/day) for 26 weeks	↓*Bacilli*, ↓*Lactobacillus*, ↓*Bacillus*, ↓*Enterococcus*, ↓*Lactococcus*, ↓*Streptococcus*	Not measured	
Ganpu tea	C57BL/6J female mice; Ganpu tea-water extract in concentration: 0.1 g/mL, 0.2 g/mL, or 0.4 g/mL every day for 3 weeks	↓*Firmicutes*; ↑*Bacteroidetes*; ↑microbial richness	↑acetic acid, ↑propionic acid, ↓butyric acid, ↓isobutyric acid, ↓valeric acid, ↓isovaleric acid	[104]
Oolong tea	C57BL/6J male mice; 0.1 g of oolong tea polyphenols per 100 g of diet for 4 weeks	↓*Firmicutes*, ↑*Bacteroidetes*, ↑*Proteobacteria*	Not measured	[84]
Liupao dark tea	Male Wistar rats with streptozotocin-induced diabetes; 150 mg/kg/day tea extract, or 30 mg/kg/day tea extract in vehicle for six weeks	↑*Clostridiales*, ↑*Ruminococcaceae*, ↑*Prevotella*, ↑*Bacteroides*, ↑*Lactobacillus*	150 mg/kg/day tea group: ↑butyric acid, ↑propionic acid, =acetic acid, ↑total SCFA30 mg/kg/day tea group: ↑butyric acid, =propionic acid, =acetic acid, =total SCFA	[105]

↑ increased compared to control group, ↓ decreased compared to control group, = no effect compared to control group, SCFA—Short-chain fatty acids.

Previous studies in laboratory animals have shown that Pu-erh tea changes the composition and functioning of the intestinal microbiota, with an increase in the relative abundance of *Firmicutes* and a decrease in the relative abundance of *Bacteroidetes*, observed in the cecum [102,106,107]. Obese mice also showed an increase in mucin-degrading *Akkermansia muciniphila*, probably caused by the stimulation of type-II and -III secretion system proteins, elongation factor Tu, and glyceraldehyde-3-phosphate dehydrogenase by polyphenols and their metabolites [102]. In studies conducted on obese rats, a significant increase in the number of bacteria negatively correlated with obesity (*Bacteroidaceae*, *Ruminococcaceae*, *Lachnospiraceae*, *Muribaculaceae*, *Rikenellaceae*), and a reduction in the number of bacteria positively correlated with obesity (*Erysipelotrichaceae*, *Lactobacillaceae*) were found [107]. In animals receiving Pu-erh tea, an increase in SCFA synthesis was also found as a result of the improvement of the composition of the intestinal microbiota [108].

Wang et al. [104] investigated the effects of Ganpu tea (Pu-erh tea + mandarin peel) on the microbiome of C57 mice. After the administration of Ganpu tea, the number of *Bacteroidetes* increased and the number of *Firmicutes* decreased, which significantly influenced the content of various types of SCFAs in the stool: an increase in the concentration of acetic and propionic acid and a decrease in butyric acid. In mice consuming Ganpu tea, a reduction in the content of branched SCFAs, mainly isobutyric and isovaleric acids, was also found. Zheng et al. [32] found that Ganpu tea increased the number of *Bifidobacterium*, *Lactobacillus* and *Lactococcus* bacteria in rats. Liupao dark tea, which is popular in China and used in traditional medicine as an antidiabetic agent, when administered to rats with streptozotocin-induced diabetes and in the diet, was found to increase the *Bacteroidetes*/*Firmicutes* ratio and the number of SCFA-producing bacteria, especially *Prevotella* and *Bacteroides* [105].

### 7.2. Herbs and Spices

Herbs and spices are widely used as traditional medications to treat diabetes and its complications. The biochemical mechanisms of the antidiabetic action of herbs and spices include the stimulation of insulin secretion from pancreatic β cells, increased glucose uptake by adipose and muscle tissue, inhibition of glucose production by hepatocytes, inhibition of intestinal glucose digestion and absorption, and regulation of the activity of certain enzymes, such as lipoprotein lipase, glucose 6-phosphatase, lactate dehydrogenase, and aldose reductase [109]. The interactions between the gut microbiota and herbs and spices mainly occur in two ways: (1) the gut microbiota breaks down active substances into more easily digestible forms, thus inducing physiological changes in the body, and (2) the active substances within the herbs and spices regulate the composition of the gut microbiota, and in turn its metabolites, cause physiological changes in the host organism [110,111]. Moreover, some of the active ingredients show bactericidal properties, e.g., ferulic acid and quercetin, while some polyphenols, e.g., ellagitannins, tannins, and chlorogenic acids, can be metabolized by intestinal microorganisms [92]. Herbs and spices provide substrates for SCFA production by the host, thus modulating the composition of the gut microbiota and regulating SCFA production [112]. The active ingredients of herbs and spices influencing the composition and activity of the microbiota are mainly oligosaccharides and other prebiotic substances, polyunsaturated fatty acids (PUFAs), phenolic compounds, anthocyanins, essential oils, and organic acids [113,114,115]. The influence of herbs and spices on the gut microbiota composition are reported in Table 2.

There are many publications in the available literature on the anti-diabetic properties of herbs used in traditional Chinese medicine. They significantly affect glucose and lipid metabolism by modulating the intestinal microbiota, especially mucin-decomposing bacteria, bacteria with anti-inflammatory properties, and lipopolysaccharide- and SCFA-producing bacteria, as well as bacteria exhibiting bile salt hydrolase activity [128]. These effects are thanks to the content of flavonoids, alkaloids, terpenoids, saponins, polysaccharides, phenylpropanoids, berberine, resveratrol, and organic acids in the herbs [111]. In rats with streptozotocin-induced diabetes, Chinese medicinal herbs were shown to increase the number of bacteria producing SCFA and anti-inflammatory compounds, and reduce the number of pathogenic bacteria associated with the diabetes phenotype, which in turn proves that the hypoglycemic mechanism of these herbs is associated with the modulation of the intestinal microbiota [120]. Berberine, present in many Chinese herbs, caused an increase in the number of butyrate-producing bacteria, mainly *Faecalibacterium* and *Roseburia*, thus reducing intestinal inflammation and lowering glucose levels in diabetic rats. An increase in the level of SCFAs in the stool was also noted [129]. The prebiotic effect of berberine has been demonstrated in numerous studies [130,131,132,133]. A previous study performed on obese mice have shown that berberine is more effective in modifying the structure of the microbiome than curcumin [131]. This study also showed that the increase in the number of *Bifidobacterium* spp. and *Akkermansia* spp. in the cecum was correlated with an improvement in intestinal barrier function and a reduction in inflammation and oxidative stress indicators in the liver [131]. Berberine indirectly supports the growth of *Akkermansia* in mice by stimulating the secretion of intestinal mucin [132]. The use of berberine in diabetic rats resulted in an increase in the number of *Bacteroidetes* and *Lactobacillaceae*, and a decrease in the number of *Proteobacteria* and *Verrucomicrobia* [134]. In contrast, in rats subjected to chronic stress, berberine increased the abundance of *Verrucomicrobia*, *Bacteroides*, *Lachnoclostridium*, *Akkermansia*, and *Anaerostipes*, and decreased the ratio of *Firmicutes* to *Bacteroidetes* by reducing the relative amount of *Firmicutes* [135]. By stimulating the growth of SCFA-producing bacteria, berberine prevents obesity and insulin resistance through the TLR4 signaling pathway [136,137].

The influence of the aqueous sacha inchi leaf extract (*P. volubilis* L.), used in China as an adjunct in the treatment of diabetes, on the composition of the intestinal microbiota in mice with streptozotocin-induced diabetes was investigated [121]. This study showed an increase in species diversity of the intestinal microbiome, especially an increase in the abundance of *Akkermansia*, *Parabacteroides*, and *Muribaculum*, and a decrease in the abundance of *Ruminiclostridium* and *Oscillibacter*. *Cyclocarya pallurus* leaves are used in traditional Chinese medicine in anti-diabetes therapy because they regulate blood glucose levels, increase insulin synthesis, and inhibit apoptosis of pancreatic β cells [138]. Flavonoids isolated from the leaves of *Cyclocarya pallurus* lower the ratio of *Firmicutes*/*Bacteroidetes* in the large intestine, which proves their probiotic effect [122,123]. Some studies have shown a beneficial effect of phenolic-rich olive leaf extract (*Olea europaea*) in experimental models of the metabolic syndrome through the modification of the gut microbiota. In a study by Vezza et al. [139], an increase in the number of *Firmicutes* and a decrease in the *Firmicutes*/*Bacteroidetes* ratio, as well as a decrease in the level of inflammatory markers, were found in diet-induced obesity mice. The use of *Anemarrhena asphodeloides* extract, at a dose of 60 mg/kg/day in rats with induced diabetes reduced the number of *Proteobacteria*, *Brachyspira*, *Facklamia*, *Klebsiella*, *Oligella*, and *Escherichia-Shigella* and increased the numbers of *Bacteroidetes*, *Actinobactercolia*, *Blautia*, and *Roseburocacteria* [125]. Oregano essential oil has antibacterial properties. It inhibits the expression of genes related to virulence in *Escherichia coli*, increases the number of *Lactobacillus*, inhibits the expression of inflammatory cytokines, and enhances the antioxidant system of the organism [140,141].

Cinnamon, ginger, oregano, black pepper, and cayenne pepper are active against *Fusobaterium*, *Ruminococcus spp.*, as well as some strains of *Clostridium difficile*, while promoting the growth of *Bifidobaterium* and *Latobacillus* [114]. In this study, cinnamon, oregano, and rosemary were active against selected *Fusobacterium* strains, while cinnamon, rosemary, and turmeric were active against selected *Clostridium* spp. The effect of the daily consumption of 5 g of mixed spices (cinnamon, Mediterranean oregano, ginger, rosemary, black, and cayenne pepper) by 29 healthy adults on the gut microbiota and SCFA synthesis was investigated, while the control group received maltodextrin [126]. The consumption of the spices resulted in a significant reduction in the number of *Firmicutes* and an increase in the number of *Bacteroidetes*. A significant negative correlation was also found between the concentration of SCFAs in the stool and the number of *Firmicutes* in the group receiving the spices, although there was no significant effect of the use of spices on the production of SCFAs. On the other hand, the average number of *Actinobateria*, *Verrucomicrobia*, *Proteobacteria*, *Fusobacteria*, *Euryarchaeota*, *Spirochaetes*, *Tenericutes*, *Cyanobacteria*, and *Lentisphaerae* was not different between groups. Turmeric and curcumin used in healthy people caused an increase in the number of *Clostridium*, *Bacteroides*, *Citrobacter*, *Cronobacter*, *Enterobacter*, *Enterococcus*, *Klebsiella*, *Parabacteroides*, and *Pseudomonas* strains and a decrease in the number of *Blautia* and *Ruminococcus* [142]. Just one serving of spices has a positive effect on the composition of the intestinal microbiota, which has been proven in studies conducted among people consuming 6 or 12 g of curry a day [116]. An in vitro study showed that curry paste increases the number of beneficial bacteria, especially *Bifidobacteria* and *Lactobacteria*, and causes an increase in SCFA synthesis, while reducing the number of *Clostridia* [117]. The main ingredient in curry powder is turmeric, and the active substance in turmeric is curcumin, which has been shown to stabilize blood glucose levels [143]. The administration of curcumin has been shown to protect against the overgrowth of opportunistic pathogens in nephropathic rats, including *Escherichia-Shigella* and *Bacteroides*, and increase the relative abundance of SCFA-producing bacteria, such as *Lactobacillus* and *Ruminococcaceae* [144]. Other studies have showed the inhibitory effect of curcumin on the growth of pathogenic strains, such as *Prevotellaceae*, *Coriobacterales*, *Enterobacteria*, and *Rikenellaceae* [145,146]. The administration of garlic to C57BL/6N mice fed a high-fat diet increased the diversity of the gut microbiome by increasing the number of *Lachnospiraceae* and reducing the number of *Prevotella* [119]. Due to the content of fructans and polysaccharides, garlic has prebiotic properties, stimulating the selective growth of beneficial bacteria, such as *Bifidobacteria*, and inhibiting pathogenic bacteria, such as *Clostridia* [147,148]. In addition to fructans, a component of garlic that favorably modulates the microbiome is allicin, which primarily increases the number of *Bifidobacterium* and *Lactobacillus* probiotic bacteria, as well as SCFA-synthesizing strains, e.g., *Blautia* [118]. In C57BL/6J mice with obesity caused by a high-fat diet, the use of black garlic melanoidins resulted in the modulation of the intestinal microbiota, with an increase in the number of SCFA-producing bacteria (*Bacteroidaceae*) and probiotic strains (*Lactobacillaceae*, *Akkermansiaceae*) and a reduction in the number of opportunistic pathogens (*Enterobacterovibrionaceae*) [149]. Cinnamon also positively modifies the microbiota of the gastrointestinal tract, mainly due to the presence of cinnamaldehyde in the essential oil, which has bactericidal and anti-inflammatory properties [133,150,151]. Cinnamon stimulates the production of SCFAs by having a positive impact on the growth of *Alloprevotella* and *Lachnospiraceae* bacteria [133]. It also stimulates the growth of beneficial bacteria *Akkermansia*, *Bacteroides*, *Clostridium* III, *Psychrobacter*, and *Intestinimonas*, and inhibits the growth of *Ruminococcus* and *Escherichia-Shigella* [151]. Cinnamon has also been found to lower blood glucose levels and prevents insulin resistance [152].

### 7.3. Probiotics, Prebiotics, Postbiotics, and Synbiotics

The main goal of the supplementation of probiotics and prebiotics in the diet is to increase the population of beneficial bacteria, eliminate pathogens, and introduce permanent changes in the composition of the intestinal flora (Table 3). 

The mechanism of action of probiotics also includes: (1) communication between microorganisms and epithelial cells, regulating the adhesion of microorganisms to epithelial cells; (2) intercellular communication between tissues, regulating intercellular adhesion; (3) as well as the regulation of signaling and transport processes of epithelial cells [153,154]. Probiotic bacteria ferment, primarily by converting sugar to lactate; however, when sugar is in short supply, the bacteria are able to alter the fermentation process and produce acetate and formate instead [155]. The acidic environment created as a result of the fermentation inhibits the growth of pathogenic microorganisms. In turn, lactic acid bacteria inhibit the growth and multiplication of pathogenic strains by secreting antibacterial metabolites: bacteriocins, lysozymes, short-chain organic acids, and β-hydroxybutyrate [156]. In the large intestine, bacteria stimulate the production of immunoglobulins and they compete for nutrients and thus prevent the adherence of pathogenic microorganisms to epithelial cells [157]. Eliminating intestinal dysbiosis with probiotics and prebiotics is associated with a reduction in the autoimmune response, a reduction in inflammation, and an improvement in intestinal integrity, through the increased expression of proteins in the intestinal epithelium [158].

Lactic acid bacteria are classified as probiotic microorganisms and belong to the phylum Firmicutes, class Bacilli and order Lactobacillales, and include over 50 genera from 6 families and over 300 species [157]. Two groups of bacteria dominate the human intestines: the Gram-positive Firmicutes (Lactobacillus spp., Bacillus spp., and Clostridium spp.) and Gram-negative Bacteroidetes [159,160]. Probiotic Lactobacillus show anti-inflammatory effects and the ability to modulate the composition of the intestinal microbiota. Studies on obese mice have shown that Lactobacillus fermentum CECT5716 restores the number of Akkermansia sp., reduces the number of Erysipelotrichi and Clostridium spp., and increases the number of Bacteroides in the microbiome [161]. The administration of the probiotic Lactobacillus plantarum, Bifidobacterium breve, and Lactobacillus fermentum to obese mice exposed to Escherichia coli increased the content of SCFAs in the stool by modifying the structure of the microbiota and reducing inflammation [162,163]. The administration of Lactobacillus acidophilus (probiotic bacteria) has been shown to alleviate the effects of obesity in mice by modulating intestinal microbiota dysbiosis and tightening the intestinal mucosa, as well as increasing the activity of Akkermansia muciniphila [164,165]. Consuming foods containing probiotic bacteria has a positive effect on the gastrointestinal microbiome [157,159]. The consumption of fermented probiotic drinks containing Lactobacillus casei Shirota (LcS) increased the number of Lactobacillus spp. and Bifidobacterium spp. in children, which was positively correlated with changes in the content of SCFAs in the stool [159]. In a previous in vitro study, a probiotic containing Lactobacillus acidophilus LA-5, Bifidobacterium animalis, and inulin caused an increase in the number of beneficial microorganisms, such as Bifidobacterium spp., Bacteroides spp., and Faecalibacterium spp., as well as an increase in SCFA synthesis [166]. The probiotic strain Bifidobacterium longum BB-46, used in healthy people, increased the number of Firmicutes (especially Lachnospiraceae and Lactobacillaceae) and Bacteroidetes in the colon, but did not affect the production of SCFAs [167]. On the other hand, the simultaneous use of Bifidobacterium longum BB-46 and lemon citric pectin stimulated an increase in the number of Faecalibacterium, Eubacterium, Lactobacillus, and Ruminococcaceae in the colon, and reduced the number of proteolytic bacteria, including Bacteroides, Clostridium, Peptoniphilus, and Streptococcus [167]. 

**Table 3:** Influence of prebiotics, probiotics, and synbiotics on the gut microbiota composition.

	Design	Effects on Gut Microbiota	Effect on SCFA Level	References
*Bifidobacterium longum* BB-46 with or without citric pectin	Probiotic strain *Bifidobacterium longum* BB-46 alone and in combination with a citric pectin from lemon on the gut microbiota using the Simulator of Human Intestinal Microbial Ecosystem	↑*Faecalibacterium*, ↑*Eubacterium*, ↑*Lactobacillus*, ↑*Ruminococcaceae*, ↓*Bacteroides*, ↓*Clostridium*, ↓*Peptoniphilus*, ↓*Streptococcus*	↑butyric acid	[167]
*Lactobacillus fermentum* CECT5716	Male C57BL/6 J obese mice; probiotic *Lactobacillus fermentum* at 5 × 10^8^ CFU in 100 μL/mouse/day for 11 weeks	↑*Bacterioidetes*, ↓*Firmicutes*, ↑*Verrumicrobia*	Not measured	[161]
*Lactobacillus acidophilus*, *Bifidobacterium longum*, and *Enterococcus faecalis*	Female C57BL/6J mice fed high-fat or high-carbohydrate diets; intragastrically administered an encapsulated probiotics (*Lactobacillus acidophilus*, *Bifidobacterium longum*, *Enterococcus faecalis*), contained at a daily dose of 2.0 × 10^7^ CFU for 30 days	↑*Bifidobacterium*, ↑*Lactococcus*, ↑*Akkermansia*, ↓*Alistipes*, ↓*Bacteroides*, ↑*Allobaculum*, ↑*Alloprevotella*, ↑*Lactobacillus*, ↑*Clostridium*, ↓*Escherichia/Shigella*	Not measured	[168]
*Lactobacillus casei Shirota*	Normal-weight and overweight school children; *Lactobacillus casei Shirota* fermented probiotic drinks (80 mL bottle) for 15 weeks	↑*Lactobacillus*, ↑*Bifidobacterium*	=acetic acid, =butyric acid, ↑propionic acid, ↑total SCFA	[159]
*Lactobacillus acidophilus* LA-5 and *Bifidobacterium animalis* BB-12	Commercial dietary supplement containing *Lactobacillus acidophilus* LA-5 and *Bifidobacterium animalis* BB-12 plus inulin (each 1 g sachet contained 9 log CFU/g of LA-5, 10 log10 CFU/g of BB-12, and 0.22 g of inulin), under simulated gastrointestinal conditions for 14 days	↑*Bifidobacterium*, ↑*Bacteroides*, ↑*Faecalibacterium*, ↑*Lactobacillus*	↓acetic acid, ↓propionic acid, ↑butyric acid	[166]
Fructans	Female nonobese diabetic T1DM NOD/LtJ mice; inulin-type fructans	↑*Ruminococcaceae*, ↑*Lactobacilli*	↑acetic acid, ↑butytic acid, ↑propionic acid	[169]
Fructans	Male diet-induced obese C57BL/6J mice; 5% cellulose (control), 10% cellulose, 10% short-chain fructo-oligosaccharides (scFOSs), or 10% inulin in diet for 4 weeks	↑*Verrucomicrobia*, ↓*Firmicutes*, ↑*Actinobacteria* (scFOS and inulin), ↑*Bacteroidetes* (inulin)	Not measured	[170]
Fructans	Patients with mild/moderately active ulcerative colitis; 7.5 g or 15 g daily oral oligofructose-enriched inulin for 9 weeks	7.5 g inulin: no effect 15 g inulin: ↑*Bacteroidaceae*, ↑*Porphyromonadaceae*, ↑*Bacteroides*, ↑*Parabacteroides*	7.5 g inulin: ↑acetic acid, =butyric acid, = propionic acid, =isobutyric acid, =isovaleric acid 15 g inulin: acetic acid, ↑butyric acid, ↑propionic acid, ↑isobutyric acid, ↑isovaleric acid	[171]
Fructans	T1DM patients aged 41–71 years consumed 16 g of inulin-type fructans (a mixture of oligofructose and inulin) for 6 weeks	↑*Bifidobacterium adolescentis*, ↑*Bacteroides ovatus*, ↑*Lachnospiraceae*, ↑*Faecalibacterium prausnitzii*, ↓*Ruminococcaceae*, ↓*Lachnospiraceae*, ↓*Erysipelotrichaceae*	↑total SCFA, ↑acetic acid, ↑propionic acid, =butyris acid, =isobutyric acid, =isovaleric acid, =valeric acid, =isocaproic acid, =caproic acid	[172]
Polysaccharides	Obese mice; 0.2% *Lycium barbarum* polysaccharides in water for 10 weeks	↓*Firmicutes*, ↑*Bacteroidetes*	=acetic acid, =propionic acid, ↑butyric acid	[173]

↑ increased compared to control group, ↓ decreased compared to control group, = no effect compared to control group, SCFA—Short-chain fatty acids, scFOSs—short-chain fructo-oligosaccharides, T1DM—Type 1 diabetes, NOD—non-obese diabetic.

Probiotics stabilize the gut microbiome and stimulate the production of SCFAs in animals exposed to xenobiotics and showing behavioral and neurodegenerative disorders [174,175,176]. In addition, probiotics can inhibit inflammation and oxidative stress by inhibiting lipid peroxidation, modulating the secretion of pro-inflammatory and anti-inflammatory cytokines, and maintaining the activity of antioxidant enzymes [177].

Postbiotics, which are products of the metabolism of living bacteria or are released from bacteria after their lysis, have beneficial health effects on the human body [178]. When used in appropriate amounts, postbiotics have been shown to promote host health and well-being, either directly or indirectly [179]. Postbiotics are identified as metabolites of probiotic bacterial strains, such as *Bifidobacterium breve*, *B. lactis*, *B. infantis*, *Bacteroides fragilis*, *Lactobacillus*, as well as *Escherichia coli* and *Faecalibacterium prausnitzii* [180]. They contain dead microorganisms and their metabolites, including enzymes, bacteriocins, peptides, lipids, teichoic acids, cell-surface proteins, polysaccharides, as well as organic acids and SCFAs [181]. The effects of postbiotics on the human body are similar to those of probiotics, however, their mechanisms of action are still unclear [182]. Postbiotics do not contain live microorganisms, therefore the risk associated with their intake is minimized, yet they are still able to modulate the intestinal microbiome. They also show immunomodulatory properties and, unlike bacteria inhabiting the intestines, they have the ability to penetrate the intestinal barrier into the GALT system and directly activate immune cells [183,184]. SCFAs are monocarboxylates produced by the gut microbiota as a result of the fermentation of polysaccharides [185]. SCFAs are involved in many metabolic processes, such as providing an energy source for microorganisms and colonocytes, controlling microbial function, fighting pathogens, protecting and maintaining intestinal integrity, and regulating immune cells and glucose metabolism [186,187,188]. Amino acid derivatives transformed by the intestinal microbiota are compounds that are potential postbiotics. Tryptophan metabolites, including tryptamine and indole-3-propionic acid, rebuild the intestinal mucosa [189]. Polysaccharide metabolites counteract oxidative stress (one of the markers of T1DM) and prevent diabetic complications [190]. They can also inhibit pancreatic α-amylase as demonstrated in in vitro studies, regulate the expression of genes involved in glucose metabolism, and increase hepatic glycogen reserves [190]. Bacteriocins and organic acids are responsible for inhibiting the growth and multiplication of pathogenic microorganisms, as well as for the maintenance of the intestinal barrier [156,191]. Postbiotics produced by *Bifidobacterium breve* and *Streptococcus thermophilus* strongly inhibit the secretion of the pro-inflammatory cytokine TNF-α, compared to other bacteria, which is indicative of specific anti-inflammatory effects of postbiotics obtained from specific bacteria [192]. This study also demonstrated the beneficial effect of commensal bacteria on intestinal homeostasis. Postbiotics also include strains of probiotic bacteria inactivated e.g., by high temperature, i.e., paraprobiotics, the health-promoting properties of which result, among others, from the immunomodulatory properties of elements of their cell walls [190]. Postbiotics obtained from various *Lactobacillus species* inhibit both Gram-positive and Gram-negative pathogenic bacteria, e.g., *Listeria monocytogenes*, *Salmonella enterica*, and *Escherichia coli* [193,194].

Prebiotics are food ingredients that have a positive effect on health and well-being by selective stimulation of the growth and/or activity of the intestinal microflora, thus also positively affecting the structure of the intestines [195]. Resistant oligosaccharides, mainly fructo-oligosaccharides and galacto-oligosaccharides, with a degree of polymerization from 3 to 9, demonstrate prebiotic activity. They form part of dietary fiber, along with the non-starch polysaccharides, lignin and cellulose [196]. Indigestible oligosaccharides have been shown to reduce inflammation in experimental colitis [197]. In this study, the enrichment of the rats’ diets with oligosaccharides resulted in changes in the microbiota, namely, an increase in *Bifidobacteria* and *Enterobacteriaceae*, and a decrease in *Clostridium* cluster IV. A higher concentration of SCFAs was also observed in the cecum of the rats. A study conducted on HLA-B27 transgenic rats with colitis showed that the use of long-chain inulin and oligofructose as a diet additive reduced the concentration of interleukin IL-1β and increased the concentration of transforming growth-factor β (TGF-β) in the cecum, as well as increased *Lactobacillus* and *Bifidobacterium* populations [198]. Fructo-oligosaccharides administered to Wistar rats with induced intestinal inflammation reduced the symptoms of inflammation [199]. This study also shows that the oligosaccharides are fermented in the upper parts of the large intestine, but their prebiotic effect extends to the distal parts of the large intestine, thus having a positive effect on inflammation in the large intestine. The administration of caramel, enriched with the prebiotic fructodisaccharides, to rats with inflammatory bowel disease, decreased the levels of pro-inflammatory cytokines TNF-α and IL-1β in the colon [200]. In female mice, inulin-type fructan promoted modulatory T-cell responses, as evidenced by an increase in CD25 + Foxp3 + CD4 + regulatory T cells, a decrease in IL17A + CD4 + Th17 cells, and modulation of the cytokine production profile in the pancreas, spleen, and large intestine [169]. Fructan was also shown to increase the *Firmicutes*/*Bacteroidetes* ratio, thereby strengthening the antidiabetogenic composition of the microbiota, as well as increasing the number of *Ruminococcaceae* and *Lactobacilli* [169]. On the other hand, *Lycium barbarum* polysaccharides increased the diversity of bacteria, decreased the *Firmicutes*/*Bacteroidetes* ratio, and improved intestinal dysbiosis caused by a high-fat diet, increasing the number of SCFA-producing bacteria *Lacticigenium*, *Lachnospiraceae*, and *Butyricicoccus*, and thus also the content of SCFAs in the feces [173]. It should be noted that the development of T1DM is associated with an abnormally increased proportion of *Bacteroidetes*/*Firmicutes*, but conflicting results are found in the literature. These data suggest that a decreased or increased proportion of *Bacteroidetes*/*Firmicutes* may be related to the obesity (more *Bacterioidetes*) or thinness (more *Firmicutes*) phenotypes [201]. Therefore, the influence of the *Bacteroidetes*/*Firmicutes* ratio remains unclear with respect to the development of T1DM, since differences in the composition of the gut microbiota may be due to the differential level of glucose in the host body fluids, which in turn may result from, e.g., diet. [158]. A commercial prebiotic containing the polysaccharides from *Acacia senegal* and *Acacia seyal*, used in an in vitro study involving an artificial intestine settled by human fecal microflora, caused an increase in the number of *Bifidobacteria* and a decrease in the number of *Clostridium histolyticum*, as well as an increase in SCFA content [202]. Some prebiotics benefit gut health by selectively stimulating gut microorganisms, including *Bifidobacteria* [203]. Enrichment of the diet with oligosaccharides in rats caused changes in the composition of the microbiota, with an increase in the number of *Bifidobacterium* and *Enterobacteriaceae*, and a decrease in the number of *Clostridium* cluster, as well as a higher concentration of SCFAs in the cecum [197]. A study conducted on HLA-B27 transgenic rats with colitis showed that the use of inulin and oligofructose in their diets reduced the concentration of inflammatory markers, and an increase in the *Lactobacillus* and *Bifidobacterium* populations in the cecum [198]. The combined use of long-chain fructo-oligosaccharides and postbiotics has beneficial effects in modulating the composition of the microbiome and immunological characteristics in early life [181]. 

Dietary fibers are edible carbohydrate polymers, with three or more monomeric units that are resistant to endogenous digestive enzymes, and therefore are not hydrolyzed and absorbed in the human small intestine [204]. However, they are fermented by gut bacteria and are the main source of carbon and energy for gut microbiota, which helps to increase gut microbiota diversity [205]. Apart from oligosaccharides, dietary fiber includes β-glucans and arabinoxylans from wholegrain cereals, pectin from fruits, vegetables and legumes, and resistant starch. After as little as 14 days of eating fiber, an increase in SCFA synthesis and an improvement in the intestinal function in adults was observed [206]. Firmicutes bacteria, Bifidobacac terium spp. and Bacteroides spp., are the main bacterial groups which degrade long-chain fiber components, e.g., resistant starch, while SCFAs are the products of anaerobic fermentation [204]. 

Synbiotics are a combination of a probiotic and a prebiotic with a synergistic effect [207]. The consumption of a combination of probiotics, prebiotics, postbiotics, and amino acids has been shown to help alleviate intestinal dysbiosis and degeneration, and thus may help improve the health of people with T1MD, and might be more effective in glycemic control than probiotics alone [208]. The beneficial effect of synbiotics on the metabolic profile of people with hyperglycemia (glycemia, biomarkers of inflammation, and oxidative stress) may result from the stimulation of the microbial production of SCFAs, which in turn leads to an increase in the concentration of glucagon-like peptide-1 (GLP-1) and inhibits hyperglycemia [177,209]. Oligosaccharides from cow’s milk were found to increase the production of acetates and lactates in the gut of human infants, leading to a lower pH [207,210]. They were also found to stimulate the growth of *Bifidobacterium* (*B. longum*, *B. breve*, *B. bifidum*, *B. pseudocatenulatum*) and enhance the efficiency of environmental colonization by these bacteria [207,210]. The administration of a synbiotic in the form of yogurt significantly reduced the development of hyperglycemia in streptozotocin-induced diabetic mice compared to the group receiving milk [211]. Synbiotic yoghurt favorably modulated the composition of the intestinal microbiota and protected the morphology of the pancreatic islets, thus preventing hyperglycemia through the microbiome-gut–pancreas axis. In diabetic mice, the administration of a synbiotic (polysaccharides isolated from *Lactobacillus plantarum* JY039 and *Lactobacillus paracasei* JY062) modified the intestinal microbiome, primarily causing an increase in the number of *Bifidobacterium* and *Faecalibaculum* and a reduction in *Firmicutes*, *Muribaculaceae*, and *Lachnospiraceae* [212]. The symbiotic also improved the functioning of other bacteria, which take part in metabolism within the intestinal tract, and it reduced inflammation by balancing the pro-inflammatory factors IL-6 and TNF-α and the anti-inflammatory factor IL-10 [212].

## 8. Perspectives 

**Figure 2:**
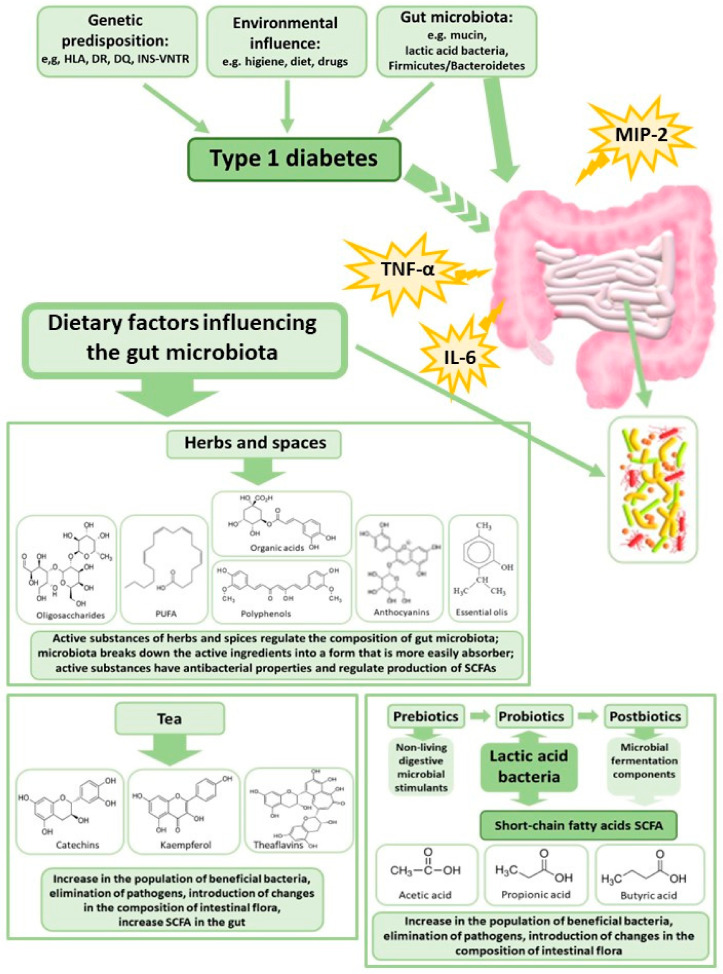
Dietary factors modulating gut microbiota in the case of type 1 diabetes. SCFA—Short-chain fatty acids, TNF-α—tumor necrosis factor-α, MIP-2—Macrophage Inflammatory Protein 2, IL-6—interleukin-6, PUFA—Polyunsaturated fatty acids.

Huang et al. [58] showed that patients with newly diagnosed T1DM exhibit a different intestinal microbiota profile associated with reduced SCFA production, as well as altered IgA indirect immunity compared to healthy subjects. These authors also showed that the intestinal microbiota of mice with T1DM induced a more intense immune response, compared to the intestinal microbiota of healthy control mice; while the administration of SCFAs to the T1MD mice decreased the secretion of IgA. This may suggest that modification of the intestinal microbiota can increase the proportion of SCFA-producing bacteria, which could in turn be effective in the prevention and/or treatment of T1DM. SCFAs are bacterial metabolites resulting from the fermentation of polysaccharides [185]. They serve as a form of communication between the microbiome and the immune system and are responsible for maintaining the balance between the anti-inflammatory and pro-inflammatory response, e.g., by signaling via free fatty acid receptors (GPRs). SCFAs also induce Treg cells by inhibiting the enzyme histone deacetylase [213]. The production of SCFAs by gut bacteria is dependent on the availability of necessary substrates. Increased daily intake of soluble and non-soluble fiber, as well as the inclusion of probiotics, prebiotics, herbs, spices, and teas that are sources of phytobiotics, in the diet, could be important in improving the composition and activity of the microbiota and thus in the prevention of metabolic disorders (Figure 2). SCFAs produced by the gut microbiota have been found to positively affect glucose metabolism in a manner which is dependent on the activation of protein kinase by adenosine monophosphate, and involves the γ receptor that is activated by peroxisome proliferators [186,187]. This mechanism seems to be crucial in diabetes development. Targeting the diet to increase the production of SCFAs may be a way to alter the immune profile, thus promoting immune tolerance, and improving glycemic control in the treatment of T1DM.

The results from previous studies suggest that there may be a diabetogenic microbiome [58,67,70,214], enabling a quick and accurate diagnosis of T1DM. Understanding how the microbiota interacts with immune cells to create immune tolerance could enable the development of new therapeutic strategies for T1DM and improve the quality of life of people with T1DM. 

It is worth noting that some studies indicate the influence of dysbiosis on the occurrence of various types of diabetes: T1DM, T2DM, and T3DM [71,215,216]. Diabetes changes the composition of the microbiota, and the altered microbiota affects the pathophysiology of the disease. Changes in the gut microbiota, in combination with a specific diet, can lead to increased intestinal permeability, which, connected with low-grade inflammation, leads to the development of insulin resistance [216]. Differences in the microbiota composition in the different types of diabetes is directly associated with the physiological symptoms of the disease. In T1DM, pathogenic microbiota cause inflammation and an autoimmune reaction, which in turn causes damage to the pancreas due to the destruction of beta cells by lymphocytes [217]. Inflammatory factors produced by an altered gut microbiota also increase the risk of developing T2DM [217]. With regards to T3DM, which is associated with Alzheimer’s disease, it has been theorized that some cyanobacteria can produce neurotoxins, leading to neurodegeneration [218]. The gut microbiota, through the gut–brain axis, modulates normal behavioral reactions related to stress and anxiety, and the vagus nerve is an important means of communication between the gut microbiota and the central nervous system [216]. However, the results of previous studies are still inconclusive and future studies need to pay more attention with regard to distinguishing specific groups of gut microbiota that are indicative of the development or prevention of T1DM, T2DM, and T3DM.

## 9. Conclusions

The analysis of the microbiota could allow for the provision of targeted probiotic therapy and individual tailored treatment. It also allows us to extend the routine diagnostics and support the doctor in a holistic approach to the patient for affected by T1DM. The manipulation of the composition of the gut microbiome and, in turn, the level of SCFAs may be a promising tool in the treatment and prevention of autoimmune diseases. On the other hand, a full understanding of the etiology of T1DM would allow for the development of preventive strategies that will avoid or delay the onset of T1DM and help maintain control over the disease after its development.

## Figures and Tables

**Table 2:** Influence of herbs and spices on the microbiota composition.

	Design	Effects on Gut Microbiota	Effect on SCFA Level	References
Curry	Healthy males; 6 g or 12 g curry mixed spices daily	↑*Bacteroides*, ↓*Bifdobacterium*	Not measured	[116]
Curry	Pasta curry with or without garcinia under simulated gastrointestinal conditions	↑*Bifidobacterium*, ↓*Bacteroides*, ↓*Clostridium*, ↓*Eubacteria*	↑lactic acid, ↑acetic acid, ↓butyric acid, ↑propionic acid, ↑total SCFAs	[117]
Garlic	C57BL/6J obese mice; (1% m/m allicin solution for drinking for 13 weeks)	↑*Bacteroidetes*, ↓*Firmicutes*, ↑*Blautia*	↑acetic acid, ↑propionic acid, ↑butyric acid, ↑isobutyric acid, ↑valeric acid, ↑isovaleric acid, ↑heptanoic acid	[118]
Garlic	C57BL/6N male obese mice; for 12 weeks	↑*Lachnospiraceae*, ↓*Prevotella*	Not measured	[119]
Chinese herbs mixture	Male Wistar rats with streptozotocin-induced diabetes; Chinese herbs (*Radix Puerariae*, *Radix Scutellariae*, *Rhizoma Coptidis*, and *Radix Glycyrrhizae*) 25 g/kg once daily by gavage for 12 weeks	↑*Flavonifractor*, ↑*Acetatifactor*, ↓*Butyricimonas*, ↓*Anaerofustis*, ↓*Butyricicoccus*, ↓*Gammaproteobacteria*	Not measured	[120]
Sacha inchi	Mice with streptozotocin-induced T1DM; Sacha inchi tea intragastrically at 400 mg kg^−1^ body weight per day for 6 weeks	↑*Akkermansia*, ↑*Parabacteroides*, ↑*Muribaculum*, ↓*Ruminiclostridium*, ↓*Oscillibacter*	Not measured	[121]
*Cyclocarya paliurus*	Male C57BL/6J obese mice; 0.1% *Cyclocarya paliurus* flavonoids in diet for 8 weeks	↑*Bacteroidetes*, ↓*Firmicutes*, ↓*Clostridiales*, ↓*Proteobacteria*, ↓*Selenomonadales*	Not measured	[122]
*Cyclocarya paliurus*	Male C57BL/6J circadian-rhythm-disorder mice; 4 weeks	↑*Prevotellaceae*, ↑*Bacteroidaceae*, ↓*Ruminococcaceae*, ↓*Lachnospiraceae*, ↓*Veillonellaceae*	Not measured	[123]
Cinnamon	Dextran sodium sulfate-induced colitis mice, 10 mg/kg or 15 mg/kg body weight cinnamon essential oil for 16 days	↓*Helicobacter*, ↓*Bacteroides*, ↑*Bacteroidales*, ↑*Alloprevotella*, ↑*Lachnospiraceae*	Not measured	[124]
*Anemarrhena asphodeloides*	Male SPF Wistar obese and diabetes (streptozotocin-induced) rats; *Anemarrhena asphodeloides* extract 20, 60, or 180 mg kg^−1^ daily by gavage for 4 weeks	↓*Proteobacteria*, ↓*Brachyspira*, ↓*Facklamia*, ↓*Klebsiella*, ↓*Oligella*, ↓*Escherichia-Shigella*, ↑*Bacteroidetes*, ↑*Actinobacteria*, ↑*Blautia*, ↑*Roseburia*, ↑*Enterococcus*, ↑*Phascolarctobacterium*	↑acetic acid, ↑propionic acid, ↑butyric acid	[125]
Mixed spices	In vitro study: bacteria isolated from human intestinal contents; cinnamon, ginger, oregano, rosemary, black and cayenne pepper, and turmeric extracts	↑*Bifidobacterium*, ↑*Latobacillus*	Not measured	[114]
Mixed spices	Healthy women and men aged 18 to 65 years; 5 g capsules of spice mixture containing 1 g cinnamon, 1.5 g oregano, 1.5 g ginger, 0.85 g black pepper, and 0.15 g cayenne pepper daily for 2 weeks	↓*Firmicutes*, ↑*Bacteroidetes*, =*Actinobateria*, =*Verrucomicrobia*, =*Proteobacteria*, =*Fusobacteria*, =*Euryarchaeota*, =*Spirochaetes*, =*Tenericutes*, =*Cyanobacteria*, =*Lentisphaerae*	=acetic acid, =propionic acid, =butyric acid, =valeric acid	[126]
Culinary herbs	Healthy women and men; cumin, garlic, onion, cinnamon, thyme, ginger, basil, rosemary, cilantro, parsley, sage, oregano, mint, dill, clove, cayenne, allspice, nutmeg, paprika, saffron, cardamom, tarragon, chives, bay leaf, coriander, red chili, black pepper, fennel seed	=*Bacteroidota*, =*Actinobacteria*, ↑*Firmicutes*, ↓*Proteobacteria*	Not measured	[127]

↑ increased compared to control group, ↓ decreased compared to control group, = no effect compared to control group, SCFA - Short-chain fatty acids.

## Data Availability

Not applicable.
